# An alternative approach for controlling bacterial pathogens in liquid and solid poultry waste using Calcium hypochlorite Ca(OCl)_2_ disinfectant-based silver nanoparticles

**DOI:** 10.1038/s41598-022-24203-8

**Published:** 2022-11-17

**Authors:** Asmaa N. Mohammed

**Affiliations:** Department of Hygiene, Zoonoses and Epidemiology, Faculty of Veterinary Medicine, Beni-Suef University, Beni-Suef, 62511 Egypt

**Keywords:** Microbiology, Environmental sciences

## Abstract

Inappropriate handling of poultry waste from the beginning to the end of the production cycle could lead to health and environmental hazards. The purpose of this study was to assess the current state of poultry waste management practices as well as to evaluate the efficacy of disinfectants (VIRKON S, Quaternary ammonium compound (QAC), Calcium hypochlorite [Ca(OCl)_2_], and nanomaterials (nano-silver particles (Ag NPs), and Ca(OCl)_2_-Ag NPs composite) on pathogenic bacteria for use in the disinfection of waste collection areas within poultry operation systems. Two hundred and ten samples were gathered from variant waste types for isolation and identification of pathogenic bacteria. Then, the efficacy of some disinfectants against fifty strains of isolated bacteria was evaluated using a broth micro-dilution assay. Results showed the most predominant bacterial isolates from wastes were *E. coli* (33.69%), *Salmonella* spp. (26.09%), followed by *K. pneumonae* (15.22%) and *L. monocytogenes* (14.13%). Ca(OCl)_2_-Ag NPs had a microbial lethal effect against all pathogenic bacteria (100%) that were isolated from liquid and solid waste. In conclusion, poultry manure waste is collected and dumped on the agricultural land around those chicken farms without any treatment. The Ca(OCl)_2_-Ag NPs composite was lethal to all pathogenic microbes isolated from waste and their collected areas at 1.0 mg/L concentration.

## Introduction

The poultry industry is one of the utmost rapidly growing agro based industries in the world today, growing at a global rate of 5% per annum*,* with its share of world meat production increasing from 15% three decades ago to 30%^1,2^. The problem that comes with the poultry operations is the waste arising, such as poultry manure, that requires to be taken care of, as non-appropriate treatment or disposal can become a health hazard for the environment and human beings^3^.

Waste from the systems used to produce poultry, including wastewater and solid waste, is produced in enormous quantities. Bedding material, dung, feed, feathers, hatchery waste, sludge and mortality are among the solid waste items. Besides, the wastewater results from the washing and disinfection process of chicken houses^4^. When poultry manure is not properly disposed of, nitrate and phosphate are released into streams, ponds, and ground water. Ammonia and hydrogen sulphide gas are also released into the atmosphere. Poultry manure may contain harmful bacteria, particularly *E. coli* and *Salmonella*. So, be sure to take precautions when handling poultry litter to minimize potential health hazards that affect both human and bird health^5,6^.

Consumers of wastewater-grown food and farmers that utilize wastewater for irrigation both risk contracting infectious diseases from the fecal coliform found in wastewater. Such diseases may consist of food poisoning, diarrhea, cholera, typhoid, dysentery, gastroenteritis, ascariasis, hepatitis and food poisoning^7^. Numerous improved methods of poultry waste handling have been proposed in an effort to lessen the threats that these wastes pose to the environment. This is because chicken wastes are known to be environmentally hazardous^8^.

Various approaches to disposing of poultry waste include burial, compositing, rendering, incineration, fertilizer, and feed for livestock. A proper way of disposing of litter and/or bird excreta is used on farm lands to enrich soil nutrient^9^. Several value-added products can be successfully made from poultry wastes, including fertilizer, power, biodiesel, animal feed, and recyclable plastic if properly managed to reduce adverse effects^3^.

Calcium hypochlorite (Ca(OCl)_2_ is a chemical disinfectant that acts as an oxidizing agent, causing damage to microbial cell walls. Because of its low cost, ease of application^10^, reaction with microbial cell membrane, and induction of cell wall lysis and bacterial death^11^, chlorine is a popular disinfectant. Quaternary ammonium compounds (QACs) are non-oxidizing disinfectants with the highest usage in the pharmaceutical sector. Through contact with phospholipids, their mode of action on the cell membrane causes cytoplasm leakage and coagulation^12^. Additionally, VIRKON S as a broad-spectrum disinfectant contains peroxygen molecules (peroxymonosulfate). It is promoted for use in human and veterinary healthcare settings as a bactericidal, virucidal, fungicidal, and sporicidal agent^13^. Nano-silver particles have strong antimicrobial activity, and their action is firmly attached to the wall of microbial cell and disrupts cell membrane permeability^14,15^. Another study discovered that filter paper impregnated with Ca(OCl)_2_ loaded nano-silver based particles had a strong biocidal effect against existing pathogenic bacteria, total viable bacteria, total and fecal coliform counts in different water sources^16^. The current work was designed to evaluate the existing state of waste management practices, identify efficient hygienic waste disposal methods, and evaluate the efficacy of some disinfectants (VIRKON S, QAC, Ca(OCl)_2_), Ag NPs, and Ca(OCl)_2_-Ag NPs nano-compounds against pathogenic bacteria isolated from liquid and solid waste from poultry farms to be used in the disinfection of manure and mortalities collected areas inside poultry buildings and to develop an acceptable management plan for minimizing the pollution hazards in poultry production sectors.

## Materials and methods

### Study the locality and period

The study was carried out in various broiler (*n* = 40) and layer (*n* = 10) poultry chicken farms scattered around the provinces of Beni-Suef and El- Faiyoum in addition to several cities such as Al-Wasta, Nasser, and Beni-Suef between June 2021 and January 2022. The chickens in broiler poultry farms were raised on a deep litter system with a stocking rate of 1.5 square feet/bird. Meanwhile, some layer chickens were kept in a battery cage system with a stocking rate of 1.7 square feet/bird.

### Ethical statement

The protocol of study design was accepted by IACUC (the Institutional Animal Care and Use Committee) of Beni-Suef University, Egypt after confirming that the welfare of animals was achieved throughout the study. Moreover, all poultry farm samples were processed in accordance with IACUC guidelines. The protocol includes study design, samples collection and size, statistical methods, and result interpretation. It was followed by a checklist of recommendations in the ARRIVE guidelines. Meanwhile, human participants were involved in the data collection, which was approved by the IRB (Institutional Review Board) of Beni-Suef University (Ref. No: IORG 0009255) after proving that the rights of human subjects were protected during their participation. Participants agreed to participate in the questionnaire data collection as voluntary participants and gave us informed consent before the study began. Consent was obtained by completion of the questionnaire, whereas consent was added in the ethical consideration of the IRB. As well, the author confirmed that all methods in the text were performed in accordance with the relevant guidelines and regulations. All data and results were recorded and statistically examined.

### Data collection

A standardized questionnaire was distributed to every farm responder in order to gather all the data and information needed to evaluate the many aspects of the poultry farms under investigation. The study focused on the biosecurity measures and farm characteristics, including the types of birds raised, the length of the cycle, the management system, the size of the farm, the type of litter used, how often it is changed during the cycle, record-keeping, types of poultry waste, and disposal techniques.

### Collecting samples

A total of 210 samples were collected from different solid and liquid waste [wastewater (*n* = 30), chick droppings (*n* = 30), chick litter (*n* = 30), feathers (*n* = 30), waste feedstuff (*n* = 30), manure collected area (*n* = 30), and mortalities collected area (*n* = 30)] inside the investigated farms for isolation and identification of different pathogenic microorganisms of health hazard on birds and surround. All samples were taken aseptically and transferred in an icebox to the lab to investigate the existence of different pathogenic microorganisms using selective media according to Roberts and Greenwood^17^.

### Isolation and identification of pathogenic bacteria from waste

The tested parameters were total viable count (TVC), fecal coliform count (FCC), and total coliform count (TCC) using the plate counting method and membrane filtration technique according to APHA^18^. Whereas the waste collected samples were diluted in 100 mL distilled water and filtered through a membrane, the membrane was incubated at 37 °C and 45 °C for 24 h for total and fecal coliform count*,* respectively using M-FC agar (EM Science, Gibbstown, NJ) and m-Endo LES agar (Difco, Sparks, MD), respectively. The enteric bacteria (*Klebsiella* spp. and *E. coli*) were isolated on MacConkey agar (CM 0115; Oxoid) and eosin methylene blue agar (CM 69; Oxoid) plates. All samples were pre-enriched on buffer peptone water and then incubated for 24 h at 37 °C to isolate *Salmonella* spp. and *Shigella* spp. Afterward, 0.1 ml of the incubated broth was added to 10 ml of Rappaport Vassilidis and incubated at 42 °C for 24 h. The samples were then streaked onto Salmonella Shigella agar (Oxoid^®^, CM 0099) and incubated for 24 h at 37 °C. Furthermore, for *Listeria monocytogene*, every 150 mL liquid sample was centrifuged for 30 min. at 5098.581065 g to separate the sediment, which was then put into a 100 mL Listeria enrichment (LEB). Each feedstuff and fecal sample (25 gm) was enriched in 225 mL of LEB and incubated at 30 °C for 48 h before being inoculated with 0.1 mL of the listeria broth onto listeria selective agar media (Palcam agar; Biokar Diagnostics, France). The inoculated plates were incubated at 37 °C for 48 h. For colony purification, all isolated bacteria were sub-cultured on nutrient agar^18^. Enteric bacteria were identified based on their colony morphology, Gram staining, and using API 20E (bioMérieux, Craponne, France). Besides biochemical tests^19^ that applied such as catalase, oxidase, TSI, methyl red and blood hemolysis tests.

### The antimicrobial activity of different disinfectants’ product

According to Amiri et al.^20^, the effectiveness of disinfectants (VIRKON S (Potassium peroxymonosulfate, Antec International TD, UK), Quaternary ammonium salts (QAC, Fluka Analytical, St. Louis, USA), and BLEACHING POWDER Ca(OCl)_2_ (a white powder and considered a strong oxidant containing 65% available chlorine) on fifty strains of different pathogenic bacteria isolated from different waste samples was evaluated using the disc diffusion method at different testing concentrations. The distilled water was used to obtain the required concentrations of different disinfectants. Sterilized Whatman filter paper was used to prepare fifty discs (disc size is10 mm**)** that were kept in screw-capped bottles. Overnight, the sterilized discs were saturated with the testing concentrations of disinfectants. Then, 100 µL of bacterial isolates (1 × 10^6^ CFU/mL) were diluted in Mueller–Hinton broth according to McFarland 0.5. Then, diluted samples (100 μL) were inoculated on plates of Mueller–Hinton agar and the discs were positioned on the agar with sterile forceps and incubated at 37 °C for 24 h. The zone of inhibition of all tested bacteria was detected through twofold serial dilution according to CLSI^21^.

#### Synthesis and characterization of nano-silver particles (Ag NPs)

Nano-silver was synthesized by a chemical reduction method according to Šileikaite et al.^22^. Ag NPs were characterized by Fourier-transform infrared spectroscopy (FT-IR) and high resolution transmission electron microscopy (HR-TEM) using a JEM100CX II transmission electron microscope (JEOL Ltd.) were used to examine and describe the microstructures and solid morphologies of nanomaterials, respectively.

#### *Ca(OCl)*_*2*_* based Ag NPs composite preparation*

Ca(OCl)_2_-Ag NPs were developed to improve disinfectant activity against the tested bacterial isolates. In a 1:1 ratio, Ag NPs at a concentration of 15 mg/L were mixed with Ca(OCl)_2_ disinfectant at both concentrations of 0.5 and 1.0 mg/L. Then, the nanocomposite was shaken well for 4 h continuously using a magnetic stirrer to avoid agglomeration and/or accumulation of nanoparticles. The Ca(OCl)_2_-Ag NPs were then centrifuged for 15 min at 5098.581065 g and washed twice with distilled water according to Ahmed et al.^23^.

#### *Biocidal efficiency of Ag NPs and Ca(OCl)*_*2*_*-Ag NPs against bacterial strains*

Fifty bacterial strains were investigated against Ag NPs at concentrations of 5, 10 and 15 mg/L besides Ca(OCl)_2_-Ag NPs at a concentration of 0.5 and 1.0 mg/L after 24 h. Exposure times using the broth macro-dilution method^24^. To 1 mL of Muller-Hinton broth (MHB), 100 mL of different freshly prepared bacterial suspensions (1 × 10^6^ CFU/mL) in normal saline were added, followed by 1 mL of Ca(OCl)_2_-Ag NPs at different concentrations (0.5 and 1.0 mg/L). Additionally, two sterilized control test tubes were utilized, one containing a bacterial inoculum and MHB and the other containing Ca(OCl)_2_-Ag NPs and Muller-Hinton broth but no bacterial inoculum. Then, 100 mL (1 × 10^6^ CFU/mL) of the tested mixture was spread on the selective agar media, incubated at 37 °C for 24 h, and investigated for the existence of microbial growth to distinguish susceptible and resistant ones to the nanocomposite. According to CLSI^21^ guidelines, susceptible strains showed no growth while resistant strains showed microbial growth on agar medium.

#### Data analysis

All data was gathered for statistical analyses using SPSS (the Statistical Package for the Social Sciences software). The obtained data from the structural questionnaire and the distribution rate of pathogenic bacterial isolates from different waste types were analyzed by the chi-square test as a non-parametric test. Besides, the anti-microbial activity of different disinfectants and silver-based nano-compounds against all bacterial isolates, Meanwhile, the one-way ANOVA test was used to analyze data on total viable count, total and fecal coliform count in liquid and solid waste arising from the investigated farms.

## Results and discussion

Poultry waste is one of the utmost imperative pollutants if not correctly disposed of. To increase the nutritional value of poultry feather wastes that can be utilized as animal feed, it is possible to chemically or biologically treatment of chicken feathers. If correctly handled to minimize negative consequences, poultry waste can be effectively used to create a variety of value-added products, such as fertilizer, biofuel, and animal feed^3^.

### The obtained data from the structured questionnaire during the survey

The main poultry operations are widely distributed in the investigated areas (deep litter and battery cage system). The number of birds reared in a deep litter system was exceeded by 20,000 birds/cycle compared to a battery cage system of 4000 birds/cycle. Twenty-eight deep litter farms (70.0%) reported having shed locked in order to segregate and/or isolate their premises for disease control, whereas five battery cage systems (50.0%) only have a locked gate around the building as shown in Table 1. Maduka et al.^25^ exhibited that the main components of biosecurity practices included fences around buildings, gates, and all in all out management represented about 80–90%. Furthermore, Mustafa^26^ recorded that the primary outlines of protection against any disease transmission are a closed gate and fence around it. Besides that, fence was not available for most farms in both semi-modern and conventional systems.

**Table 1:** The obtained data of structured questionnaire disseminated to the investigated poultry farms.

Questionnaire data	Deep litter system (*n* = 40)	Battery cage system (*n* = 10)
Type of bird species	Broilers and layers	Broilers and layers
Number of birds reared/cycle	10,000- ≥ 20,000	1000- ≥ 4000
Shed lock/gate presence	28 (70.0)	5 (50.0%)
Footbath dip at entry gate of building	23 (57.5)	7 (70.0% )
Isolation of sick birds in separated area	31(77.5)	8 (80.0%)
**Type of litter used**		
Sawdust	15 (37.5)	Not applied
Wood shaving	25 (62.5)	
**Methods of litter/manure dispose during cycle**		
Clear all	6 (15.0%)	10 (100.0%)
Remove 10 cm from top layer	34 (85.0)	0.0 (0.0%)
**Frequency of litter change**		
Once/week	27 (67.5%)	Not applied
Every month	13 (32.5%)	
**Poultry mortalities disposal option**		
Disposal landfill	10 (25%)	0.0 (0.0%)
Burial	8 (20%)	3.0 (30%)
Incineration	16 (40%)	5.0 (50%)
Disposal in waterway	6 (15%)	2.0 (20%)
Disinfection in between cycle	31 (77.5%)	7 (70.0%)
Mortality rate/cycle	5 (12.5%)	1 (10.0%)

The obtained data of disseminated questionnaire in poultry systems are significantly different at *P* < 0.05.

Furthermore, at the entry gate of the building, application of footbath dip is presented at 70% in the battery cage system compared to 23 out of 40 (57.5%) in the poultry farms of the deep litter system in this study. Haftom et al.^27^, found that at the gate, footbaths were used by 80% of the broiler poultry farms, while 88% of the farms practiced washing and disinfecting their buildings and equipment. Ali et al.^28^ indicated that a high level of biosecurity was applied in the closed system than in the open system, whereas 84.6% was used in the footbath dip at the entrance of the shed. On the contrary, the isolation rate of sick birds in separated areas was similarly high in both investigated systems, at 77.5 and 80%, respectively, to avoid dissemination of highly pathogenic diseases. Sudarnika et al*.*^29^ found that twenty-four poultry farmers segregated sick birds from healthy birds at 96% and disposed of them by burning or burying them. Meanwhile, just two poultry farms left dead birds thrown away at 4.4%. Furthermore, Mohammed and Helal^30^ found that most respondents pointed out that they isolated the diseased chicks in a chosen area by using the same building as the rest of the flock. In addition, hygiene stations were rarely present in some poultry premises, and the absence of biosecurity plans employed on-farm was concerning.

In a deep litter system, the litter types that used were sawdust and wood shavings at 37.5 and 62.5%, respectively. For litter disposal during the cycle, some broiler poultry farms clear all litter (15.0%), while 85.0% of these farms remove 10 cm from the top layer and add another. On the other hand, in a battery cage, manure is disposed of during cycle 100% in a tray that is far away from the birds and then collected in manure areas. The frequency of litter change in deep litter was 67.5% once/week while other farms were at 32.5% every month. The methods of disposal of poultry mortalities were incineration followed by disposal in landfill and burial, especially in deep litter (40.0, 25.0 and 20.0%), while in battery cages, incineration and burial were the most applied methods of mortality disposal (50.0 and 30.0%, respectively). Mohammed and Helal^30^ stated that participants in each poultry operation system clarified lack of capital and sufficient space for applying hygienic measures of disposal of dead birds that involve burning or burial. Besides, poultry producers did not apply composting as a safe method to dispose of dead birds. Furthermore, the risk of environmental degradation and disease transmission is increased when poultry carcasses are dumped in waterways or on a road where dogs might find them and scavenge. Moreover, Muduli et al.^3^ reported that strict monitoring of the burial of dead birds and/or mortalities on the farm is required to avoid contamination of groundwater sources; additionally, composting could be used to reduce bacterial pathogens and then recycled as soil fertilizer. In the current text, mortalities disposed in waterways were 20% in battery cages as compared to 15.0% in deep litter systems. Disinfection in between cycles was available in both systems, whereas 77.5% in the deep litter system and 70% of the battery cage applied. Finally, the poultry producers reported that the mortality rate/cycle was significantly greater in deep litter (12.0%) than in battery cage (10%) at P 0.005 as presented in (Table 1**)**. Turkson and Okike^31^ mentioned that to prevent and control highly pathogenic diseases such as HPAI H5N1, the application of biosecurity measures is a critical point. Additionally, the majority of small-scale broiler chicken farms employ minimal or no biosecurity controls, which may raise the likelihood of disease transmission between poultry farms, mortality rates, and the danger of exposing people to potential health risks^32^.

### The distribution pattern of pathogenic bacteria from liquid and solid waste

The frequent distribution of pathogenic microbes arising from investigated farms in Table 2 clarified that 87.62% (184/210) of the total examined samples positively contained highly pathogenic bacteria. The most predominant bacterial isolates from waste were *E. coli* (33.69%, 62/184), *Salmonella* spp. (26.09%, 48/184), followed by *K. pneumonae* (15.22%, 28/184), and *L. monocytogenes* (14.13%, 26/184). Meanwhile, *Shigella flexneri* was detected in the least percentage (10.87%, 20/184). The highest percentage of *E. coli *was isolated from chicks dropping (46.43%, 13/28), manure collected area (40%, 12/30), and wastewater (37.04%, 10/27) followed by mortalities collected area (32.14%, 9/28) and chicks’ litter (30%, 9/30). Oppositely, *Salmonella* spp. was recorded in the highest percentage in wastewater (37.04, 10/27) and chicks dropping (28.57%, 8/28) followed by mortalities collected area (25%, 7/28) whilst *K. pneumonae* was isolated at a higher rate from mortalities collected area (21.43%, 6/28) followed by both chicks’ litter and manure collected area (16.67%, 5/30 each). Furthermore, *L. monocytogenes* was highly isolated from waste feed (27.78%, 5/18) and feathers (17.39%, 4/23). Besides, *Shigella flexneri* was also detected in feathers (21.74%, 5/23) and chicks’ litter (13.33%, 4/30). These findings support those of Sahoo et al.^33^ who showed that managing poultry litter had a significant impact on the health of birds. Keeping the chicks' litter dry is another essential aspect of managing chicken farms. In the presence of elevated litter pH and moisture content, Soliman et al.^34^ explained that chicks' litter is a favorable medium for bacterial growth and transmissions like *S*. *Typhimurium*. Additionally, Tiweri et al.^35^ noted that *L. monocytogenes* was frequently found in the vicinity of animals and persisted for an extended period of time in animal waste, soil, water, and feed. According to Abdel-Latef and Mohammed^36^, contamination of the poultry environment by highly pathogenic bacteria is the main reason for greater death rates and large economic losses in these farms. Environmental contamination may be caused by bird fecal droppings reflecting less stringent hygiene practices in poultry farms.

**Table 2:** Frequent distribution of pathogenic bacteria isolated from liquid and solid waste arising from investigated farms.

Waste type	Total examined No	Positive No. (%)	Frequent distribution of Pathogenic bacteria (%)				
			*E. coli*	*K. Pneumonae*	*Salmonella* spp.	*Shigella* flexneri	*L. monocytogene*
Chicks dropping	30	28 (93.33)	13(46.43)	3 (10.71)	8 (28.57)	2 (7.14)	2 (7.14)
Chicks’ litter	30	30 (100)	9 (30.0)	5 (16.67)	7 (23.33)	4 (13.33)	5 (16.67)
Feathers	30	23 (76.67)	5 (21.74)	3 (13.04)	6 (26.09)	5 (21.74)	4 (17.39)
Wastewater	30	27 (90.0)	10 (37.04)	4 (14.81)	10 (37.04)	1 (3.70)	2 (7.41)
Waste feed	30	18 (60.0)	4 (22.22)	2 (11.11)	5 (27.78)	2 (11.11)	5 (27.78)
Manure collected area	30	30 (100)	12 (40.0)	5 (16.67)	5 (16.67)	3 (10.0)	5 (16.67)
Mortalities collected area	30	28 (93.33)	9 (32.14)	6 (21.43)	7 (25.0)	3 (10.71)	3 (10.71)
Total	210	184 (87.62)	62 (33.69)	28 (15.22)	48 (26.09)	20 (10.87)	26 (14.13)

The chi-square association of frequent distribution of bacterial isolates in examined samples is statistically significant at (χ^2^) = 96, *P* < 0.05.

### The total bacterial count and indicator microorganisms isolated from liquid and solid waste

The total viable count and indicator bacteria that were identified from liquid and solid waste that the deep litter system produced were displayed in Table 3. It was discovered that the TVCs in both mortalities and manure collected areas were significantly greater (8.21 × 10^7^ ± 1.2 × 10^5^ and 7.32 × 10^7^ ± 2.3 × 10 CFU/100gm) followed by chicks’ litter (6.71 × 10^7^ ± 3.5 × 10 CFU/gm) and wastewater (3.56 × 10^7^ ± 1.1 × 10^5^ CFU/mL) compared with its count in feathers and waste feed (2.34 × 10^4^ ± 1.1 × 10 and 2.34 × 10^5^ ± 1.1 × 10^5^ CFU/gm, respectively). In addition, TCCs were isolated at the highest rates in chicks’ litter and manure collected areas (900 ± 1.1 and 900.0 ± 4.8 CFU/100gm), whilst in waste feed it was 110.0 ± 6.2 CFU/100gm. As well, FCCs were significantly high in both chicks’ litter and manure collected areas (350.0 ± 4.1 and 350.0 ± 3.0 CFU/100gm, respectively) followed by chicks dropping (220.0 ± 1.2 CFU/100 gm) and wastewater (220.0 ± 2.2 CFU/100 mL). Meanwhile, FCCs in feathers and waste feed did not exceed 60.0 ± 3.6 and 90.0 ± 1.1 CFU/100 gm, respectively. Abd El-Salam et al.^37^ found that the wastewater contains 1600 colonies of total coliform. Hartel et al*.*^38^ pointed out that the possible source of fecal coliforms is fresh poultry litter, and the composting process of litter can principally eradicate these bacteria. Nevers et al*.*^39^ clarified that fecal contaminations including livestock, poultry, and other fecal wastes are potential sources of bacterial pathogens with human health risks in recreational waters. Zhuang et al.^40^ noted that chicken farms are a crucial source of fecal pollution in the environment as poultry excrement contains bacteria that are harmful to the environment and humans.

**Table 3:** Total viable count, total and fecal coliform count in liquid and solid waste arising from investigated farms.

Waste type	Total examined No	Positive No. (%)	TVCs	Indicator microorganisms	
				TCCs	FCCs
Chicks dropping	30	28 (93.33)	7.23 × 10^6^ ± 2.2 × 10^5a^	350 ± 3.2^b^	220.0 ± 1.2^b^
Chicks’ litter	30	30 (100)	6.71 × 10^7^ ± 3.5 × 10^4ab^	900 ± 1.1^a^	350.0 ± 4.1^a^
Feathers	30	23 (76.67)	2.34 × 10^4^ ± 1.1 × 10^3b^	90.0 ± 5.0^c^	60.0 ± 3.6^c^
Wastewater	30	27 (90.0)	3.56 × 10^7^ ± 1.1 × 10^5^	240 ± 3.4^ab^	220.0 ± 2.2^b^
Waste feed	30	18 (60.0)	2.34 × 10^5^ ± 1.1 × 10^4b^	110.0 ± 6.2^c^	90.0 ± 1.1^ab^
Manure collected area	30	30 (100)	7.32 × 10^7^ ± 2.3 × 10^5a^	900.0 ± 4.8^a^	350.0 ± 3.0^a^
Mortalities collected area	30	28 (93.33)	8.21 × 10^7^ ± 1.2 × 10^5a^	350.0 ± 2.5^b^	240.0 ± 5.1^b^

*TVCs* Total viable counts (CFU/mL; CFU/gm), *TCCs* Total coliform counts (CFU/100 mL; CFU/100gm), *FCCs* Fecal coliform counts (CFU/100 mL; CFU/100 gm), *CFU* Colony forming unit.

The mean values of TVCs, TCCs, and FCCs in different waste types (mean ± SE) of different superscript letter^(a,b,c & ab)^ of the same column are significantly different at *P* ≤ *0.01.*

### Characterization of Ag NPs and Ca(OCl)_2_-Ag NPs using TEM and FT-IR

**Figure 1:**
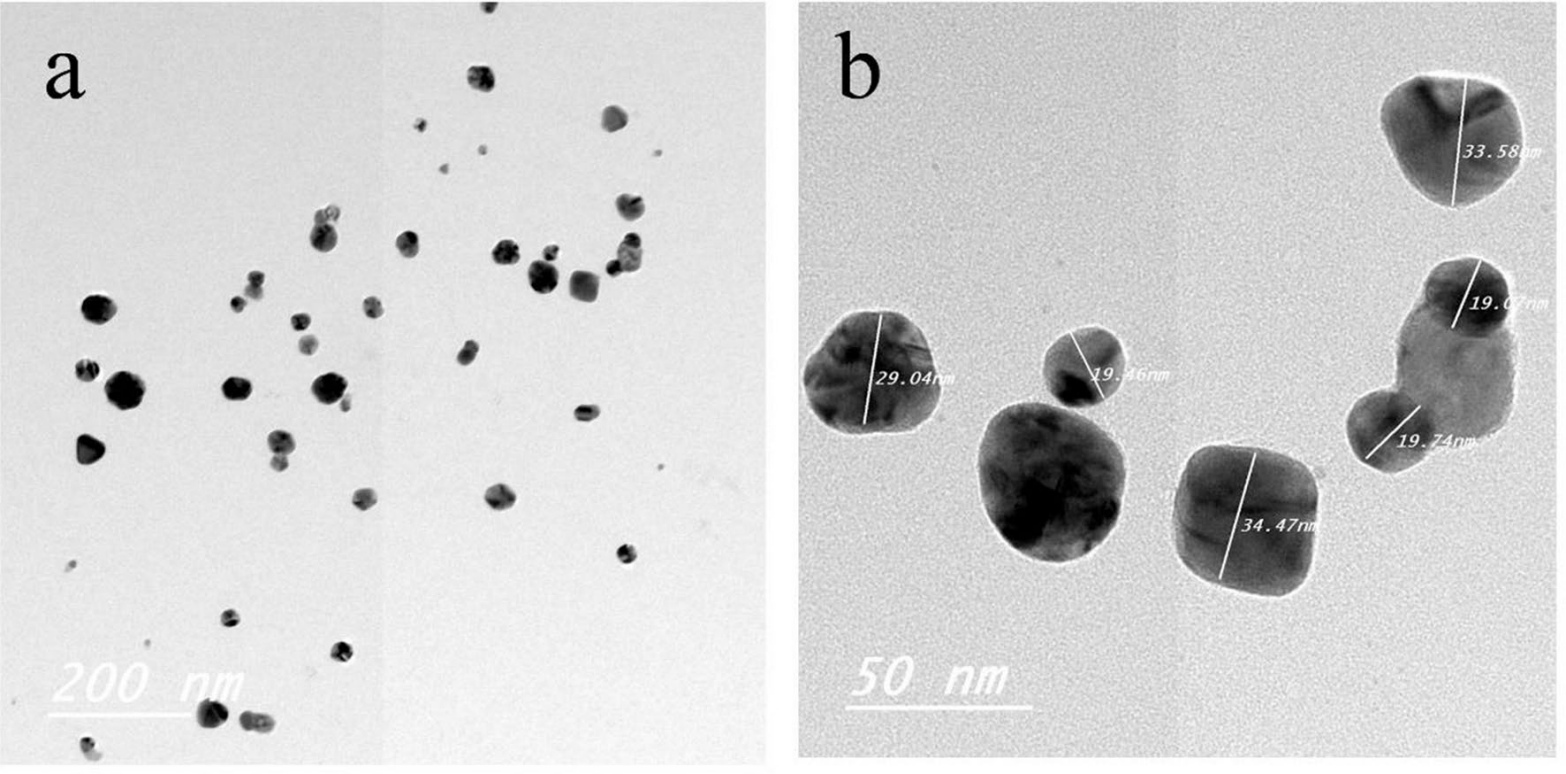
TEM photography of nano-silver particles (Ag NPs). The morphological shape (**a**) showed the fine spherical and elliptical particles of nano-silver besides the diameter of NPs (**b**) was ranged from 19.07- 34.47 nm.

**Figure 2:**
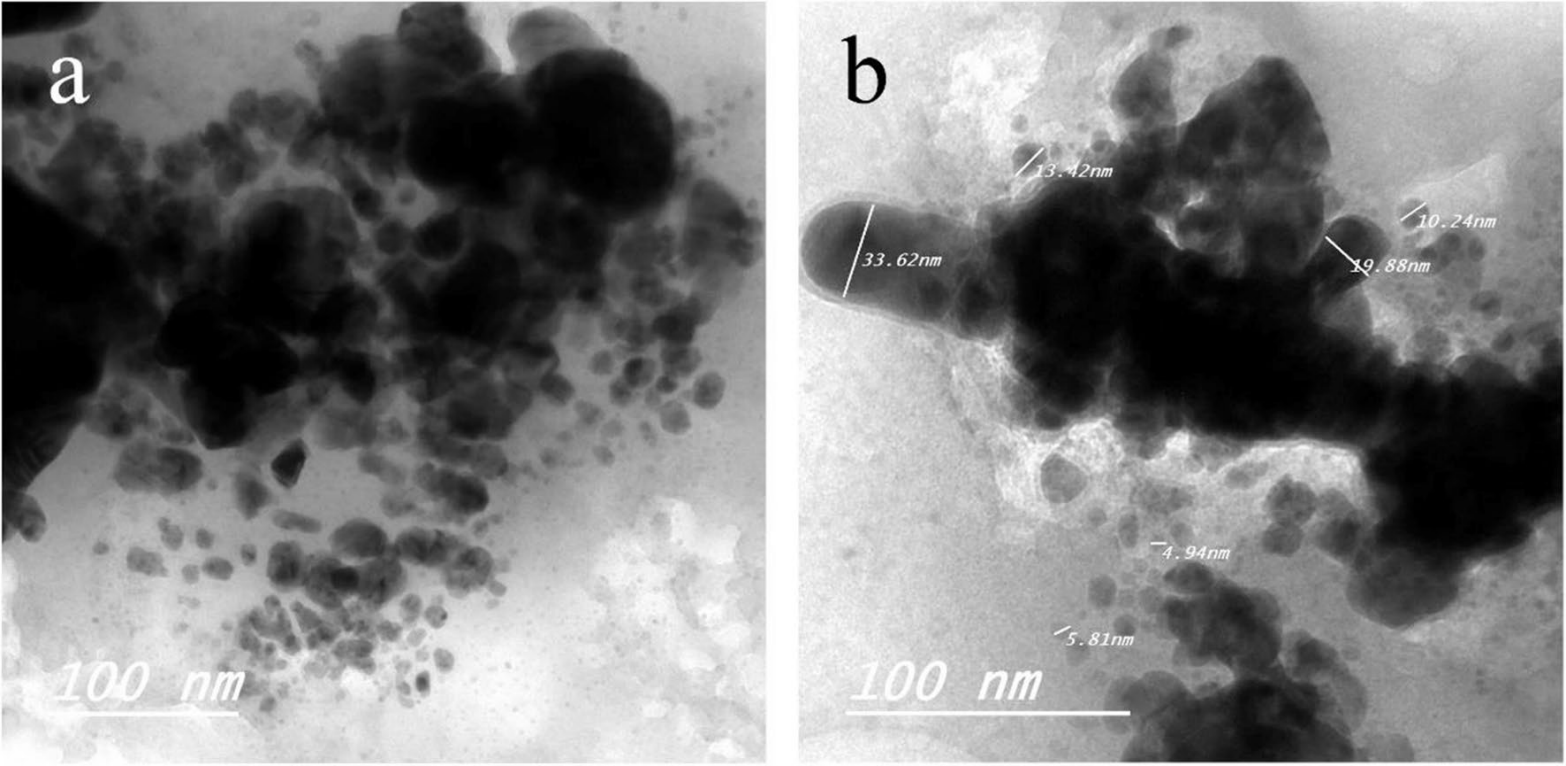
TEM photography of Ca(OCl)_2_ loaded on Ag NPs (**a**-**b**). The morphological shape displayed the spherical and elongated nanoparticles (NPs) of composite (**a**). Besides, the diameter of the NPs (**b**) ranged from 4.94 to 33.62 nm.

**Figure 3:**
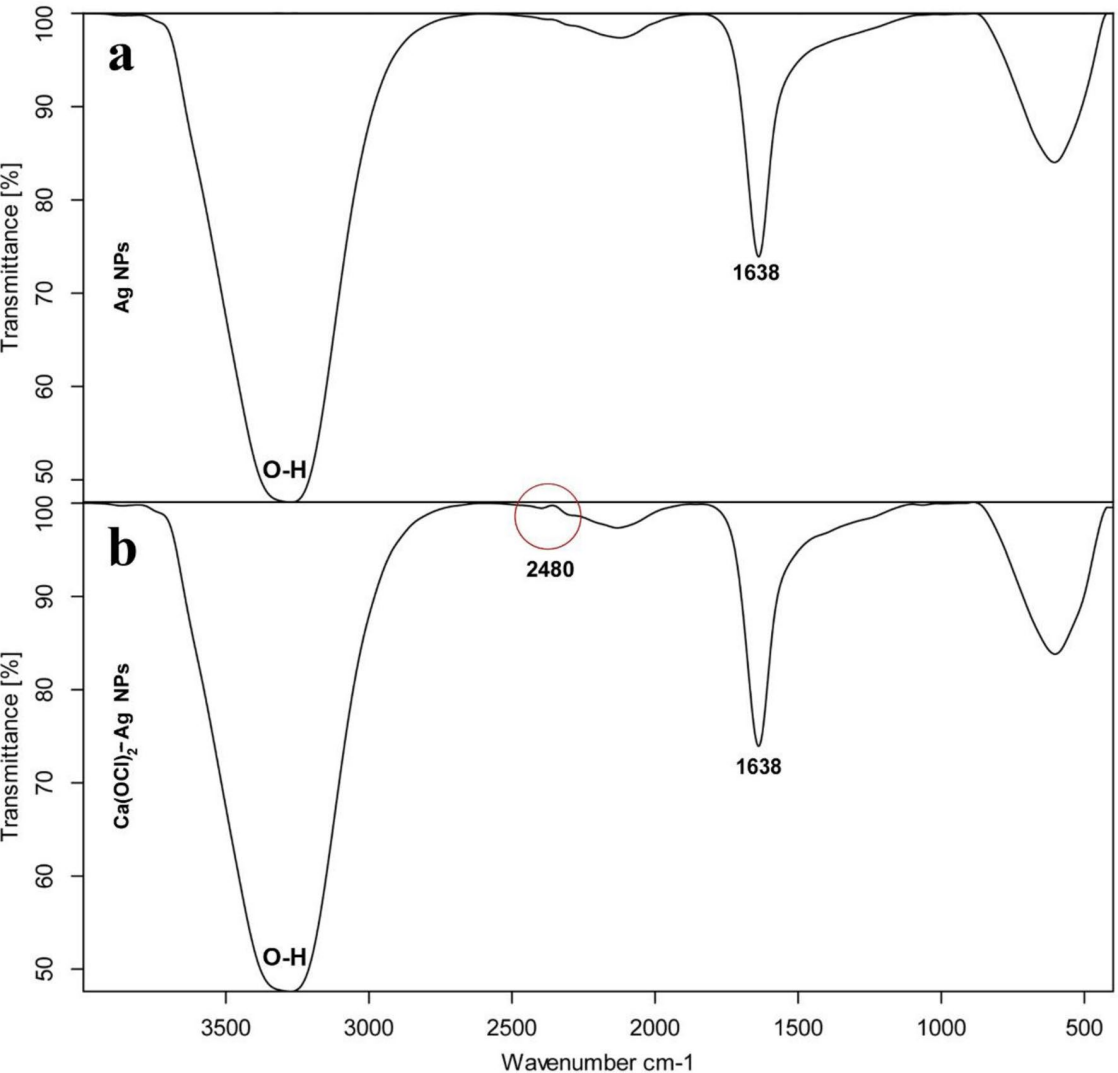
FT-IR spectrum of Ag NPs (**a**) and Ca(OCl)_2_-AgNPs (**b**).

TEM of Ag NPs showed the morphological shape (spherical and elliptical) and the size of nano-silver particles ranged between 19.07–34.47 nm (Fig. 1a,b). TEM photography of Ca(OCl)_2_-AgNPs revealed the spherical and elongated morphological shape of the composite's nanoparticles (NPs). Besides, the diameter of the NPs ranged from 4.94 to 33.62 nm (Fig. 2a,b). Ag NPs **(**Fig. 3a) showed specific peaks at 3272.18, 1638.07, 919.01 and 604.61 cm^−1^. Furthermore, FT-IR of Ca(OCl)_2_-AgNPs (Fig. 3b) showed characteristic peaks at 3273.57, 2132.25, 1638.21 and 602.51 cm^−1^, confirming the successful loading of Ca(OCl)_2_ on the Ag NPs. Roy et al.^41^ pointed out that the FT-IR spectra of nano-silver particles exhibited the characteristic peak of Ag NPs that is located at 1638 cm^−1^. In addition, Mohammed^16^ displayed FT-IR of Ca(OCl)_2_ loaded on Ag NPs whereas a specific peak appeared at 2480 cm^−1^, approving the loading in a successive way.

### The antimicrobial activity of disinfection compounds, Ag NPs and Ca(OCl)_2_-Ag NPs

Pathogenic bacteria were isolated from several waste types and their susceptibility to the disinfection products, Ag NPs and Ca(OCl)_2_-Ag NPs (Table 4) revealed that the susceptibility of all isolated bacteria to VIRKON S was not greater than 70% at the highest concentration of 2% after 24 h of exposure when compared to the lowest concentration, whereas their susceptibility was between 30–60%. As well, the susceptibility of isolates to quaternary ammonium compounds was not exceeded by 80%, except that *L. monocytogene* was highly sensitive at 100% at a concentration of 1.5 mg/L. Møretrø et al.^42^ clarified that due to the presence of resistance genes, *L*. *monocytogenes* was tolerant to sub-lethal concentrations of QAC.

**Table 4:** Biocidal efficacy of tested disinfectants product and nanocomposite against isolated bacteria from waste types.

Tested compound	Susceptibility pattern (%) of bacterial isolates to disinfectants and nanoparticles. No. of tested isolates (n = 50 strains)											*P value*
	Tested conc. (mg/L)	*E*. *coli*		*K*. *Pneumonae*		*Salmonella* spp.		*Shigella* flexneri		*L*. *monocytogene*		
VIRKON S	0.5%	S	R	S	R	S	R	S	R	S	R	0.05
		40	60	50	50	40	60	50	50	30	70	
	1.0%	60	40	60	40	50	50	60	40	50	50	
	2.0%	70	30	60	40	60	40	70	30	60	40	
QACs	0.5	30	70	60	50	40	60	70	30	50	50	0.04
	1.0	50	50	70	30	60	40	70	30	70	30	
	1.5	70	30	80	20	80	20	80	20	100	0.0	
Calcium hypochlorite Ca(OCl)_2_	0.5	20	80	30	70	40	60	40	60	50	50	0.06
	1.0	60	40	80	20	70	30	90	10	80	20	
	1.5	90	10	90	10	90	10	90	10	100	0.0	
Ag NPs	5	30	70	50	50	40	60	60	40	30	70	0.001
	10	40	60	70	30	60	40	80	20	50	50	
	15	70	30	90	10	80	20	100	0.0	90	10	
Ca(OCl)_2_-Ag NPs	0.5	80	20	70	30	60	40	90	10	80	20	0.03
	1.0	100	0.0	100	0.0	90	10	100	0.0	100	0.0	

*S* Susceptible, *R* resistant.

Ortiz et al.^43^ clarified that there is a positive association between frequent use of a QAC disinfectant and the existence of *L. monocytogene* resistant to it which might be attributed to the presence of resistance genes to QAC disinfectants^44^. In this context, *L*. *monocytogene* was significantly more sensitive to Ca(OCl)_2_ (100%), followed by *K. pneumonae*, *Salmonella* spp., and *Shigella flexneri*, which were 90% sensitive at 1.5 mg/L (*P* ≤ 0.05). Yim et al.^45^ discovered that Ca(OCl)_2_ and QAC were more effective than sodium hypochlorite at completely eliminating vegetative cells and spores. Oppositely, in this study, all bacteria exhibited resistance profiles to Ag NPs that exceeded 30% at concentrations of 5.0 mg/L at 24 h of exposure times compared to the highest concentration of 15 mg/L where the susceptibility of isolates was exceeded 80% for *L. monocytogene* and *k. pneumonae.* Furthermore*, Shigella flexneri* was 100% sensitive. Belluco et al.^46^ concluded that the overdue effect of Ag NPs on the pathogenic bacteria might have been caused by the slow release of silver ions from the Ag NPs. The effectiveness of Ca(OCl)_2_-Ag NPs against pathogenic bacterial isolates was investigated in the current study, which found that bacterial isolates (*E. coli, K. Pneumonae, Shigella Flexneri,* and *L. monocytogene*) from various waste types were highly sensitive (100%) to Ca(OCl)_2_-AgNPs at a concentration of 1.0 mg/L after 24 h of exposure. Salmonella spp. were 90% sensitive to Ca(OCl)_2_-AgNPs at the lowest concentration of 0.5 mg/L.

Silver ions' ability to bind to Ca(OCl)_2_, penetrate bacterial cell membranes, and improve membrane permeability may be responsible for this action, confirming that the biocidal activity demonstrated by Ca(OCl)_2_-Ag NPs is synergistic. These results are consistent with those reported by Morones et al.^47^ and Sondi and Salopek-Sondi^48^, who found that employing Ag NPs to treat water increased cell membrane permeability and leakage of the cytoplasm of *E. coli*. Additionally, Ag NPs have been shown to have an antimicrobial effect is credited with the release of Ag ions from the Ag NPs surface and binding on thiol groups in membrane proteins, resulting in bacterial enzymatic systems are inhibited and DNA aggregation^49,50^. Mohammed^16^ found that the microbial effect of Ag NPs against *E. coli* and *S. aureus* was exceeded by 80%, whilst it has a lethal effect against *K. pneumoniae* (100%) at the highest concentration (5.0 mg/L) after exposure time (180 min).This could be due to Ag ions' ability to bind to and infiltrate the microbial cell membrane. Furthermore, Dilarri et al.^51^ demonstrated that the Ca(OCl)_2_ mechanism of action targets the microorganism’s cytoplasmic membrane, which may be responsible for cell death.

## Conclusions

Monitoring of microbial contaminants in different waste types (liquid and solid) arising from poultry operation systems could be helpful in the hygienic disposal of these wastes and alleviate their hazardous nature in the environment. All pathogenic bacteria isolated from liquid and solid waste from the tested poultry farms were killed (100%) by Ca(OCl)_2_-Ag NPs when used at a concentration of 1.0 mg/L that confirmed the improvement of Ca(OCl)_2_ disinfectant power throughout its loading on nano-silver based particles. Ca(OCl)_2_-Ag NPs’ ability to penetrate microbial cell membranes and subsequently impede growth is thought to be the cause of their bactericidal effects. Furthermore, using Ca(OCl)_2_ and/or Ca(OCl)_2_-Ag NPs nanocomposite to disinfect manure and mortalities collected areas could destroy all pathogenic bacteria and alleviate the environmental hazards of microbial contamination.

## Data Availability

All data are included in the main manuscript and are freely accessible.
